# Benralizumab response in adult-onset Still’s disease with eosinophilic complications and associated lung disease: DRESS or biologics-induced cytokine imbalance?

**DOI:** 10.1016/j.ero.2026.100229

**Published:** 2026-09-07

**Authors:** Onur Akyol, Lukas Jörg, Urs Christian Steiner, Beatrice Mosimann, Christine Bernsmeier, Christoph T Berger, Thomas Daikeler, Diego Kyburz, Florian Kollert

**Affiliations:** Department of Rheumatology, University Hospital Basel, Basel, Switzerland; Department of Allergology and Immunolgy, Inselspital, Bern University Hospital, University of Bern, Bern, Switzerland; Department of Rheumatology and Immunology, Inselspital, Bern University Hospital, Bern, Switzerland; Department of Clinical Chemistry, Inselspital, Bern University Hospital, University of Bern, Bern, Switzerland; Department of Obstetrics, University Hospital of Basel, Basel, Switzerland; University Centre for Gastrointestinal and Liver Diseases, University Hospital Basel, Basel, Switzerland; University Centre of Immunology, University Hospital Basel, Basel, Switzerland; Department of Rheumatology, University Hospital Basel, Basel, Switzerland; Department of Rheumatology, University Hospital Basel, Basel, Switzerland; Department of Rheumatology, University Hospital Basel, Basel, Switzerland; Department of Rheumatology and Immunology, Inselspital, Bern University Hospital, Bern, Switzerland

Dear Editor,

We report a woman with adult-onset Still’s disease (AOSD) who developed eosinophilic complications and lung disease (LD) during interleukin (IL)-1/IL-6 receptor (IL-6R) inhibitor therapy. We complemented this case with a retrospective analysis that highlighted the challenges of applying the Registry of Severe Cutaneous Adverse Reactions (RegiSCAR) criteria for the diagnosis of drug reaction with eosinophilia and systemic symptoms (DRESS) in patients with AOSD.

A 36-year-old pregnant woman in her second trimester presented with fever, maculopapular salmon-pink rash, lymphadenopathy, and anaemia. AOSD was diagnosed, and initial treatment with prednisone and anakinra resulted in clinical improvement. Elevated transaminases prompted a switch to tocilizumab. The patient achieved remission and delivered a healthy infant. Several weeks postpartum, she presented with a new flare. Tocilizumab was replaced with canakinumab. Within a few days after the first dose of canakinumab, she developed fingertip exfoliation, a new type of more diffuse, generalised widespread erythema, eosinophilia, nausea, vomiting, and dyspnoea. Imaging demonstrated pulmonary oedema, septal thickening, and serositis. Skin biopsy, performed 8 days after canakinumab treatment, confirmed eosinophilic dermatitis. A DRESS-like reaction was suspected (RegiSCAR score 8/9, ie, definite DRESS), and methylprednisolone pulse therapy was started.

Calcineurin inhibition and a single 30-mg subcutaneous dose of benralizumab resulted in normalisation of radiological abnormalities and eosinophilia (Fig 1A). Months later, eosinophilia recurred but improved after an additional dose of benralizumab and has remained absent. Furthermore, reintroduction of cytokine inhibition was possible without reoccurrence of eosinophilic complications (IL-1 and IL-6R inhibitors); currently the patient is in remission under tacrolimus, anakinra and low-dose prednisone. Furthermore, lymphocyte transformation testing (LTT) for tocilizumab, anakinra and canakinumab was negative; however, the test was performed under oral prednisone. There was no evidence for viral reactivation (including Epstein-Barr virus [EBV], cytomegalovirus [CMV], human herpesvirus [HHV]-6, HHV-7, and HHV-8).

**Figure 1:**
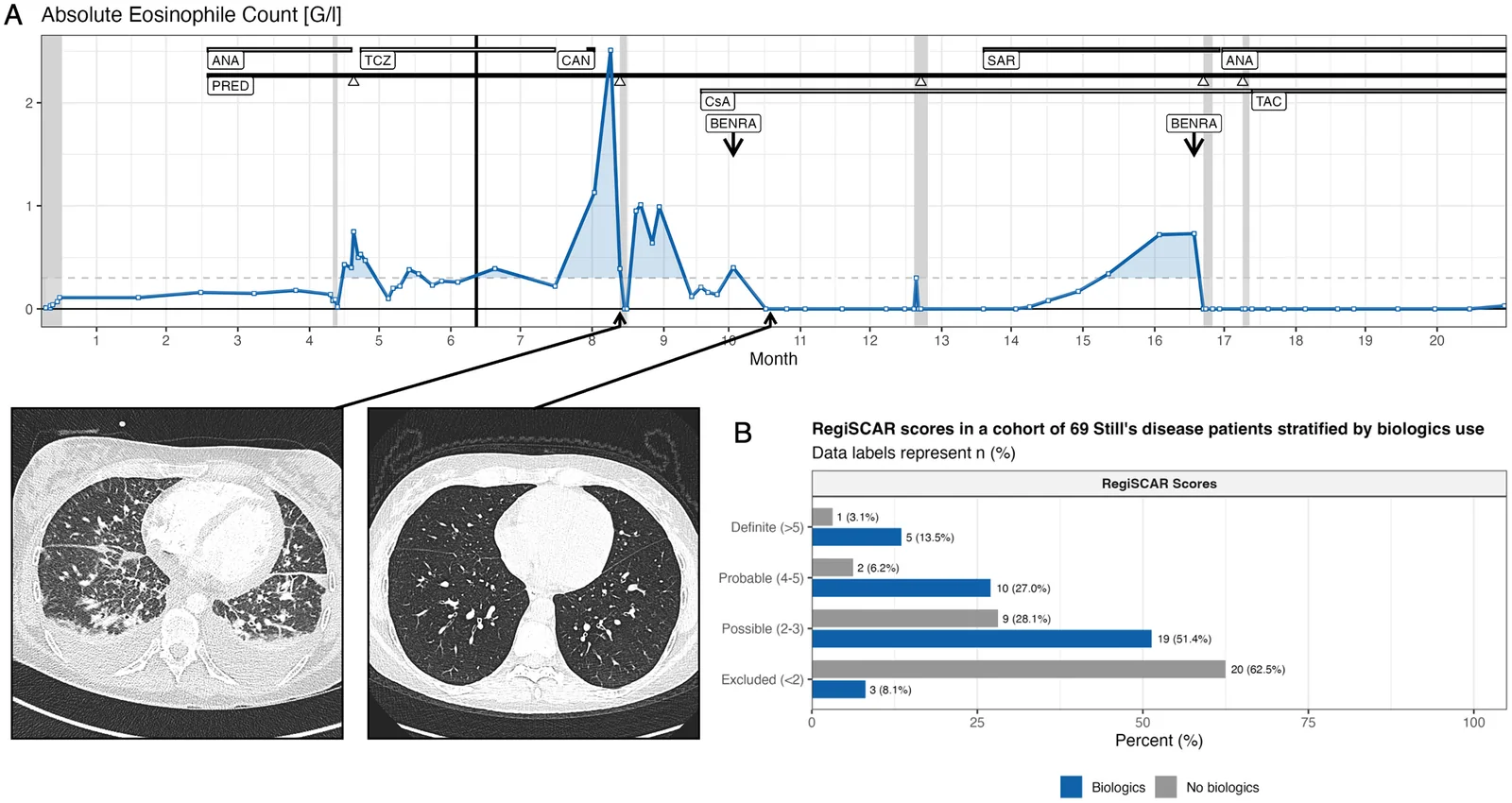
Clinical course and retrospective analysis of Registry of Severe Cutaneous Adverse Reactions (RegiSCAR) criteria in adult-onset Still’s disease. (A) Clinical course of the patient with eosinophilic complications and Still’s disease-associated lung disease during interleukin (IL)-1/IL-6 receptor (IL-6R) inhibitor therapy. Horizontal bars indicate periods of medication exposure. Triangles denote intravenous methylprednisolone pulse therapy, and grey shaded areas represent hospitalisations. The vertical black line indicates delivery of a healthy infant. The chest computed tomography scan demonstrated septal thickening, interstitial oedema, and bilateral pleural effusions, with complete radiological resolution following initiation of calcineurin inhibition and benralizumab. (B) Proportion of patients with adult-onset Still’s disease fulfilling RegiSCAR criteria-based drug reaction with eosinophilia and systemic symptoms (DRESS) classification criteria cumulatively over the disease course and according to ever receiving biologic treatment (IL-1 or IL-6R inhibition). ANA, anakinra; BENRA, benralizumab; CKN, canakinumab; CsA, cyclosporine A; PRED, prednisone; SAR, sarilumab; TAC, tacrolimus; TCZ, tocilizumab.

Our initial interpretation of the case was guided by recent reports of an emerging, high-mortality Still’s disease-associated LD in paediatric patients treated with IL-1/IL-6R inhibitors and linked to eosinophilic complications and HLA-DRB1*15, leading to the hypothesis of an underlying severe delayed hypersensitivity reaction consistent with DRESS [1,2]. However, alternative concepts propose that these manifestations instead reflect cytokine plasticity, where altered cytokine patterns due to therapeutic blockade influence immune cell differentiation [3]. The distinction between classical DRESS and a biologics-induced cytokine imbalance syndrome has important therapeutic implications, as a diagnosis of DRESS directly affects decisions regarding discontinuation or re-exposure to highly effective cytokine-targeted therapies.

To investigate the potential diagnostic overlap between AOSD and DRESS, we retrospectively applied the RegiSCAR criteria to 69 patients treated for AOSD at the University Hospital Basel between 2000 and 2026, assessing the criteria cumulatively over the disease course and considering each criterion positive if it occurred at any time after disease onset. Remarkably, two-thirds of patients fulfilled criteria for at least ‘possible’ DRESS, with this proportion exceeding 90% among patients ever receiving biologic therapy (IL-1/IL-6R inhibitors) (Fig 1B). Even atypical lymphocytosis, a hallmark feature of DRESS, was observed in 22% of biologic-treated patients (Supplementary Table S1).

These observations demonstrate substantial overlap between AOSD manifestations and current DRESS classification criteria and suggest that RegiSCAR criteria-based classification alone may not reliably distinguish classical drug hypersensitivity from severe systemic inflammation or biologics-induced cytokine imbalance in AOSD. In addition, they may also affect interpretation of previous RegiSCAR criteria-based studies linking IL-1/IL-6R inhibition to Still’s disease-associated LD and HLA-DRB1*15 [2].

Our case further supports the concept that eosinophilic complications in AOSD may represent a potentially treatable inflammatory phenotype. The negative LTT, the short onset after switching to canakinumab, together with the absence of eosinophilic complications and LD recurrence following reintroduction of cytokine inhibition, makes classical drug hypersensitivity less likely and instead points towards a cytokine imbalance-driven mechanism. To the best of our knowledge, this is the first reported case of Still’s disease-associated LD with eosinophilic complications successfully managed with IL-5R blockade.

## Funding

This research did not receive any specific grant from funding agencies in the public, commercial, or not-for-profit sectors.

## CRediT authorship contribution statement

**Onur Akyol:** Writing – original draft, Methodology, Formal analysis. **Lukas Jörg:** Writing – review & editing, Methodology, Conceptualization. **Urs Christian Steiner:** Writing – review & editing, Methodology. **Beatrice Mosimann:** Writing – review & editing. **Christine Bernsmeier:** Writing – review & editing. **Christoph T Berger:** Writing – review & editing. **Thomas Daikeler:** Writing – review & editing. **Diego Kyburz:** Writing – review & editing. **Florian Kollert:** Writing – review & editing, Writing – original draft, Methodology, Conceptualization.

## Competing interests

FK reports a relationship with F. Hoffmann-La Roche, Novartis, UCB, NovoNordisk, Pfizer, Gilead that include equity or stocks, speaker fees, consulting or advisory, funding grants, and travel reimbursement. DK reports relationships with AbbVie, Janssen, Novartis, UCB, Lilly, Pfizer, Johnson & Johnson, and Sanofi that include speaker fees and consulting or advisory. BM reports relationships with MEDICA and Roche, including speaker fees and funding grants. LJ reports relationships with GSK and AstraZeneca that include consulting or advisory. TD reports relationships with AbbVie, Novartis and Sanofi that include speaker fees, funding grants and consultancy or advisory. US reports relationships with Otsuka, BioCryst, GSK, Sanofi and Takeda that include consultancy or advisory and speaker fees. If there are other authors, they declare that they have no known competing financial interests or personal relationships that could have appeared to influence the work reported in this paper.

## Appendix Supplementary materials

Image, application 1
