# Supplementary material for: Benralizumab response in adult-onset Still’s disease with eosinophilic complications and associated lung disease: DRESS or biologics-induced cytokine imbalance?
Source: EULAR Rheumatol Open. 2026 Sep 7;2(3):100229. doi: 10.1016/j.ero.2026.100229 (PMC13578616; doi:10.1016/j.ero.2026.100229)
Supplement: Supplementary file 1 [file mmc1.docx]

**Supplementary Table 1:** Cumulative RegiSCAR scores in a cohort of 69 Still's disease patients stratified by biologics use (ever treated with IL-1/IL-6R inhibitors)

|  | **Total**  **n (%)** | **Biologics**  **n (%)** | **No biologics**  **n (%)** |
| --- | --- | --- | --- |
| **RegiSCAR score** | 69 | 37 | 32 |
| Exluded (<2) | 23 (33.3%) | 3 (8.1%) | 20 (62.5%) |
| Possible (2-3) | 28 (40.6%) | 19 (51.3%) | 9 (28.1%) |
| Probable (4-5) | 12 (17.4%) | 10 (27%) | 2 (6.2%) |
| Definite (>5) | 6 (8.7%) | 5 (13.5%) | 1 (3.1%) |
| **RegiSCAR items** |  |  |  |
| Acute skin eruption | 44 (63.8%)) | 30 (81%) | 14 (43%)) |
| Fever ≥38.5% | 58 (84%) | 36 (97.3%) | 22 (68.7%) |
| Lymphadenopathy | 25 (36.2%) | 16 (43.2%) | 9 (28.1%) |
| Internal organ involvement | 32 (46.4%) | 25 (67.6%) | 7 (21.9%) |
| Eosinophilia | 14 (20.3%) | 10 (27%) | 4 (12.5%) |
| 0.7-1.49 G/l | 13 (18.8%) | 9 (24.3%) | 4 (12.5%) |
| ≥1.5 G/l | 1 (1.4%) | 1 (2.7%) | 0 |
| Atypical lymphocytes | 12 (17.4%) | 8 (21.6%) | 4 (12.5%) |
| Resolution time >15d | 39 (56.5%) | 29 (78.4%) | 10 (31.3%) |
| Absence of signs of alternative diagnosis | 46 (66.6%) | 27 (73%) | 19 (59.3%) |
